# *Klebsiella pneumoniae* liver abscesses: pathogenesis, treatment, and ongoing challenges

**DOI:** 10.1128/iai.00508-24

**Published:** 2025-07-03

**Authors:** Michelle Angeles-Solano, Zajeba Tabashsum, Liang Chen, Sarah E. Rowe

**Affiliations:** 1 Department of Microbiology and Immunology, University of North Carolina-Chapel Hill 318275, Chapel Hill, North Carolina, USA; 2 Division of Clinical and Translational Therapeutics, School of Pharmacy and Pharmaceutical Sciences, University at Buffalo 15497, Buffalo, New York, USA; University of Pittsburgh, Pittsburgh, Pennsylvania, USA

**Keywords:** *Klebsiella* liver abscesses, antibiotic treatment failure, hypervirulent *Klebsiella pneumoniae*, multidrug resistance, antibiotic tolerance

## Abstract

Over the past 30 years, a distinct pathotype of hypervirulent *Klebsiella pneumoniae* (hvKp) has emerged, characterized by its ability to cause severe tissue-invasive infections, including liver abscesses in otherwise healthy individuals. *Klebsiella* liver abscesses (KLA) are most prevalent in East and Southeast Asia; however, their global incidence is rising, with hvKp now recognized as an important pathogen in Europe and the United States. While *K. pneumoniae* often colonizes the gut asymptomatically, KLAs develop when hvKp disseminates from the gut to the liver via the portal vein. Strains expressing K1 and K2 capsule types demonstrate the highest resistance to clearance by Kupffer cells, the liver resident macrophages, and are responsible for most KLA cases. KLAs present as fibrously encapsulated lesions composed of bacteria, pus, and immune cells. Treatment typically involves a combination of abscess drainage and antibiotic therapy; however, clinical outcomes are often poor, even in the absence of antibiotic resistance. This is due, in part, to significant barriers to achieving effective antibiotic efficacy within abscesses that can result in devastating complications such as metastatic infection, liver resection, or death. The emergence of KLA caused by multidrug-resistant hvKp strains, although still rare, represents an additional and alarming therapeutic challenge. This review explores the pathogenesis of KLA and highlights critical obstacles to effective management and therapy.

## INTRODUCTION

*Klebsiella pneumoniae* is a Gram-negative, non-motile encapsulated bacteria, and strains are separated into two pathotypes: classical *K. pneumoniae* (cKp) and hypervirulent *K. pneumoniae* (hvKp) (1). CKp strains are frequently multidrug resistant and are a major cause of hospital-acquired infections, such as pneumonia, bacteremia, and urinary tract infections (UTIs), particularly in immunocompromised individuals (2). HvKp can also cause these types of infections but are distinct in their ability to cause severe community-acquired tissue-invasive infections in immunocompetent individuals (1). While hvKp infections are most prevalent in populations in Asian countries, their incidence is rising globally, and hvKp is now recognized as a significant cause of pyogenic (*pus-filled*) liver abscesses in the United States and Europe (3).

*K. pneumoniae* is a common colonizer of the gastrointestinal (GI) tract*,* detected in 4%–35% of stool samples in Western countries and in 18%–88% of individuals in endemic regions (4–8). In Asian populations, approximately 10% of individuals colonized with *K. pneumoniae* harbor strains that are likely hvKp (8). However, the prevalence of hvKp GI carriage in Western countries remains poorly defined, as most studies do not distinguish between cKp and hvKp strains.

KLAs can occur when hvKp migrates from the gut to the liver via the portal vein, typically following bowel leakage (9, 10). However, KLAs can also arise from other primary sites, such as lung or wound infections (11). Gut colonization by hvKp is also thought to facilitate community transmission through the fecal-oral route (10).

Once established, hvKp liver abscesses are difficult to treat with antibiotics and are prone to metastatic spread, leading to conditions such as endophthalmitis or meningitis that can result in vision loss or death, respectively (12, 13). Reported mortality rates for KLAs range from 5% to 40% (9, 14–16). Despite the significant clinical impact of KLAs, the mechanisms underlying the poor outcomes are not well understood. In this review, we explore the pathogenesis of KLAs, and the challenges associated with management and treatment of these infections.

## BACTERIAL FACTORS ASSOCIATED WITH KLA

Compared to cKp, hvKp is equipped with additional virulence factors that promote community-acquired infections in healthy individuals. This section provides a brief overview of these virulence determinants, reviewed in more depth previously (17–21).

All *K. pneumoniae* strains are encapsulated, with 77 distinct capsule (K) types identified through phenotypic studies and over 150 capsule synthesis loci (K-loci, KL) characterized by genome sequencing (22). Capsule is a thick polysaccharide layer that surrounds the bacterial cell and functions in immune evasion (23). Wzc, an inner membrane tyrosine kinase, plays a central role in capsule polysaccharide synthesis and export (24, 25). Through cycles of phosphorylation and dephosphorylation, Wzc regulates both the polymerization of the capsule and its export across the bacterial envelope. This dynamic control ensures proper capsule chain length and effective translocation, both of which are essential for maintaining the thick, protective capsule associated with virulence, and immune evasion in *K. pneumoniae* (26, 27). HvKp strains typically possess a thicker, more abundant capsule (hypercapsule) and display a hypermucoid phenotype (also called hypermucoviscous/HMV) (1). Hypercapsule production and HMV are related but distinct features, with HMV characterized by longer and more uniform polysaccharide chains, providing additional protection from the immune response (26, 28). Phenotypically, the HMV phenotype is tested in the lab using the string test, where a colony is stretched with a loop to form a filament ≥5 mm long, indicating a positive result (12, 28). In contrast, capsule production is frequently assessed by measuring uronic acid levels, as many capsular polysaccharides contain uronic acid, making it a surrogate marker for quantifying capsule production (29). Although HMV production is highly correlated to the hvKp pathotype, not all hvKp strains display this phenotype (12, 18). However, HMV production is highly associated with KLA cases (26, 30–32).

The plasmid-borne genes that encode RmpADC and RmpA2 (Regulator of mucoid phenotype) are key virulence factors that contribute to hypercapsule production and the HMV phenotype (18, 28). RmpA functions as an autoregulatory factor, activating the expression of the *rmpADC* operon. RmpC promotes hypercapsule production by stimulating capsule biosynthesis gene expression, and RmpD drives the HMV phenotype (26, 28, 33). Specifically, RmpD binds to Wzc which regulates capsule polymerization and export. This interaction leads to the production of more consistent, longer polysaccharide chains, thereby leading to the HMV phenotype (34). RmpA2, which shares approximately 80% identity with RmpA, plays a similar role in enhancing capsule production (28, 35). It is important to note that both the hypercapsule and HMV phenotypes depend on the capsule biosynthesis pathway. Defects in capsule synthesis genes (e.g., *ΔwcaJ*, *ΔmanC*, *Δwzc*, and *Δwzy*) result in capsule-null and non-HMV phenotypes and are associated with reduced virulence (26, 36). In contrast, loss of HMV does not necessarily equate to loss of capsule (37).

*K. pneumoniae*, like all pathogens, need to acquire iron within the host to sustain growth. CKp strains often produce two siderophores, enterobactin (encoded by *ent* operon) and yersiniabactin (*ybt*), that facilitate iron acquisition. While enterobactin is ubiquitous in nearly all *K. pneumoniae* strains and broadly conserved across the *Enterobacteriaceae* family, yersiniabactin is present in only a subset of strains and is strongly associated with clinical isolates and increased pathogenicity (38–40). HvKp strains have acquired one or more additional siderophores, aerobactin (*iuc*) and salmochelin (*iro*), reviewed recently (21). Acquisition of these additional siderophores by hvKp is thought to increase virulence through enhanced ability to scavenge iron, particularly in iron-restricted environments (41).

Although the liver is the major site of iron storage in the body, the host restricts iron availability to pathogens during infection through a defense mechanism known as nutritional immunity (42, 43). Nutritional immunity involves the production of iron-binding proteins such as transferrin and lactoferrin, as well as hepcidin, a hormone that decreases iron absorption in the liver (43). Additionally, lipocalin-2 (Lcn2), also known as neutrophil gelatinase-associated lipocalin (NGAL), is an innate immune protein that plays a critical role in host defense by binding sideophores (44, 45). By sequestering enterobactin, Lcn2 prevents the bacteria from re-importing the iron-loaded siderophore, effectively starving them of this essential nutrient and inhibiting their growth. Salmochelin (a glycosylated form of enterobactin), yersinobactin, and aerobactin evade Lcn2 binding, allowing continued iron acquisition despite the host’s immune response (46–48). Although these siderophores have not been experimentally demonstrated to be essential for KLA, they are considered key virulence factors for hvKp strains that typically cause KLAs (18).

## EPIDEMIOLOGY

Until the 1980s, *Escherichia coli* was the leading cause of pyogenic liver abscesses (PLAs) worldwide. In 1986 in Taiwan, hvKp was first identified as the cause of several cases of liver abscesses and septic endophthalmitis (49). Since then, the prevalence of KLAs has increased and now accounts for up to 80% of PLAs in East and Southeast Asia (3, 50, 51). In North America and Europe, hvKp is estimated to cause ~10%–20% of PLAs, but incidence is also rising in these regions (30, 51–58).

### Surveillance

*K. pneumoniae* strains are classified into sequence types (ST) based on variations in housekeeping gene sequences (19, 22, 59, 60). These STs are used to track their lineage as well as virulence and resistance traits. CKp strains increasingly display multi-drug resistant (MDR) phenotypes, such as the production of extended-spectrum beta β-lactamases (ESBLs) or carbapenemases (60). High-risk MDR clones, such as those from ST258 and ST307, are frequent causes of nosocomial infections and hospital outbreaks (61, 62). Although these strains exhibit low virulence in animal models (63), their multidrug resistance poses a significant threat to immunocompromised patients, with mortality rates in patients reaching as high as 50% (64).

The dominant lineage of hvKp is ST23, which is responsible for approximately 80% of KLA cases (59, 65). HvKp strains are traditionally susceptible to almost all classes of antibiotics, except ampicillin due to chromosome encoding narrow-spectrum β-lactamase SHV gene (1). Despite the drug susceptibility displayed by these strains *in vitro*, treatment failure of KLAs is common, and this will be discussed in detail in a later section.

In contrast to many Asian countries, routine clinical tests to differentiate hvKp from classical strains, such as the string test, are not standard practice in Europe and North America (66, 67). This may indicate that the incidence of hvKp infections is underreported in these regions. In support of this, a retrospective clinical study investigating the prevalence of HMV-positive isolates in cases of community-acquired *K. pneumoniae* bacteremia in Calgary between 2001 and 2007 found that 8.2% of *K. pneumoniae* isolates were HMV positive by string testing (68). Statistical analysis revealed that the HMV phenotype was strongly linked to the presence of the *rmpA* gene and to infections involving the liver and central nervous system (68). Additional retrospective studies in Europe and North America identified hvKp as the causative agent of invasive infections, including liver abscesses (52, 68–70). In a hospital system in Western Pennsylvania, 4.8% of respiratory *K. pneumoniae* isolates were positive by the string test, indicating that strains with hypervirulent features are contributing to respiratory infections in the United States (71). However, the extent to which hvKp contributes to urinary tract and surgical site infections remains unknown.

More recently, convergent strains that display the antibiotic-resistant traits of cKp as well as hypervirulent traits of hvKp have emerged (72–76). These strains represent an enormous clinical challenge due to the limited antibiotic options available for treating the invasive infections they cause. In 2024, the World Health Organization (WHO) and the European Center for Disease Prevention and Control issued warnings about the growing threat of carbapenem-resistant hvKp strains and recommended routine testing to facilitate diagnosis and disease management (66, 67). Notably, cases of drug-resistant hvKp strains causing KLA have been reported, including instances with fatal outcomes (77–79). Regardless of the resistance profile, hvKp strains can cause severe tissue-invasive infections, underscoring the importance of prioritizing surveillance efforts for both convergent and antibiotic-sensitive hvKp strains.

Genotypic markers offer a precise and objective method for identifying and characterizing hvKp (28, 80, 81) but may overestimate the presence of hvKp. Recent data from a large multicentered study in China reveal a significant shift in hvKp epidemiology, with 91% of infections now nosocomial or healthcare-associated, and 43.1% of isolates exhibiting multidrug resistance (82). However, it is noted that hvKp is defined by the presence of some combination of *peg-344*, *iroB*, *iucA*, *rmpA*, or *rmpA2*, and therefore, it is unclear how many of these strains are phenotypically hypervirulent. The string test remains a low-cost and straightforward method for detecting HMV, but it can yield false negatives due to environmental conditions that modulate HMV expression, potentially underestimating hvKp prevalence (83, 84). Sedimentation resistance assays are more quantitative and reliable, but similarly affected by growth conditions, highlighting the need for careful standardization when identifying HMV positive clinical isolates (85–88). Despite these efforts (28, 80, 81), genotypic prediction of hvKp remains challenging, underscoring the necessity for further research that integrates both genotypic and phenotypic data from globally representative strains to improve accuracy and understanding.

## CLINICAL MANIFESTATION & PATHOGENESIS

When bacteria enter the liver sinusoids, Kupffer cells play a primary role in preventing infection by eliminating infiltrating pathogens (89). The K1 and K2 capsule types have been shown to be most resistant to clearance by Kupffer cells in animal studies (23, 90). These findings correlate with the clinical observation that KLAs are almost exclusively caused by K1 or K2 serotypes (28, 91, 92). However, cases involving other capsule types have also been reported (79, 93). Recent animal models have shown that *K. pneumoniae* liver abscesses are preceded by a phase in which the bacteria resist macrophage-mediated clearance, occasionally replicating intracellularly (90). It is thought that early evasion is followed by bacterial escape from Kupffer cells, triggering a neutrophil influx that fails to control the infection, leading to widespread distribution of extracellular bacteria throughout the evolving abscess (90).

KLAs typically occur in the absence of hepatobiliary disease and, unlike most other pyogenic liver abscesses, are almost always monobacterial infections (94–96). The most common clinical presentation of KLAs includes persistent fever, chills, and right upper quadrant tenderness or abdominal pain (94). Additionally, blood work from KLA patients frequently shows elevated levels of liver enzymes (alanine aminotransferase [ALT], aspartate aminotransferase [AST], alkaline phosphatase [ALP]), leukocytosis, increased C-reactive protein levels, and reduced albumin levels (94–97). However, KLAs can occasionally present with minimal or atypical symptoms, leading to delayed diagnosis until the infection has significantly advanced. For instance, a case report described a 68-year-old male, with no history of diabetes or immunosuppression, presented to an outpatient hospital in Japan complaining of lower back pain, weakness in his lower limbs, and slurred speech (98). An abdominal computed tomography (CT) scan later revealed a liver abscess in the right lobe (98). In another case study from the United Kingdom, a middle-aged man presented with loss of appetite and painless vision loss. The patient had no fever or abdominal pain but was diagnosed with KLA and endophthalmitis that resulted in permanent vision loss (99). These studies emphasize the importance of recognizing atypical presentations in clinical practice to prevent delays in detection that could allow the infection to progress to life-threatening stages.

The highly vascular nature of the liver facilitates bacterial entry into the bloodstream. Once in circulation, the bacteria can spread to distant sites, such as the eyes through the blood-retina barrier, to cause endophthalmitis (100, 101). The retina and choroid are richly supplied with blood vessels and provide a route for bacterial colonization (100, 101). The eye is an immune-privileged site with limited immune surveillance. In this environment, infections can progress rapidly, and without prompt and aggressive treatment, patients are at risk of permanent vision loss (102). The bacteria can also spread to the central nervous system, causing meningitis and brain abscesses (103–105). In patients with KLA, metastatic infection occurs in 11%–37% of cases (94, 106–108), highlighting the need for early diagnosis and intervention. Notably, such metastatic complications, particularly endophthalmitis and meningitis, are rarely observed in infections caused by cKp and are strongly associated with hvKp (1).

## RISK FACTORS

While KLAs frequently occur in immunocompetent individuals, certain host factors increase the risk of developing these infections. Diabetes mellitus is a primary risk factor for KLA, but not for liver abscesses caused by other organisms (94–96, 109, 110). The link between diabetes and KLA is unclear, but neutrophils from patients with poor glycemic control show reduced phagocytosis of *K. pneumoniae* strains with K1 and K2 capsule types compared to non-diabetic patients, which could potentially aid initial infection (111). Fatty liver disease has also been more strongly associated with KLA compared to liver abscesses caused by other pathogens (110). Further studies are needed to clarify how these co-morbidities affect hvKp pathogenesis.

The higher prevalence of cases among individuals of Asian descent in endemic regions is generally attributed to colonization patterns rather than specific genetic predispositions to KLA (9). Reports from Western countries often associate KLA cases in individuals of Asian descent with prior travel or immigration from endemic areas (3, 57, 112–114), supporting the idea that regional hvKp exposure, rather than genetic ancestry, is the primary factor.

Antibiotic use may elevate the risk of developing KLA, as suggested by both clinical and experimental evidence. A retrospective study in Taiwan found that recent ampicillin or amoxicillin therapy (within the past 30 days) significantly increased the likelihood of KLA (115). Complementary animal studies demonstrated that administering ampicillin to hvKp-colonized mice heightened their susceptibility to liver abscess formation (115). Another complementary animal study demonstrated that antibiotic treatment altered the host microbiota and resulted in a temporary increase in hvKp shedding in the feces (116). This study suggests that antibiotic treatment could increase host-to-host transmission of hvKp through the fecal-oral route. However, stool colonization does not always correspond to the strains found in liver abscesses. For example, a Korean study revealed that only 27% of K1 and K2 KLA cases had matching strains in stool and abscess samples (117), suggesting that KLAs may also originate from sources other than the gut. In contrast, studies suggest that gastrointestinal carriage of cKp is a major risk factor for subsequent infection, where up to 45%–80% of infecting strains are typically found in the gut (118, 119). The Korean study was limited by its sample size (37 patients) and focus on a specific geographic population (117), but it nonetheless highlights the possibility of extraintestinal sources. Supporting this, emerging evidence indicates that environmental reservoirs, such as contaminated water, soil, sewage, and food, could also serve as a source of exposure (120–123).

## INTERVENTION

### Standard of care

KLAs are commonly identified using a CT scan or ultrasound imaging combined with *K. pneumoniae* culture from abscess aspirate or blood (13). Treatment involves systemic antibiotic therapy, which is customized based on the results of *in vitro* antibiotic susceptibility testing. In addition to antibiotics, drainage of the liver abscess is often recommended although the location of the abscess can significantly impact the patient’s risk (124). For instance, a study examining six case reports of patients with KLAs found that five of the patients successfully underwent percutaneous drainage. In some cases, abscesses located beneath the diaphragm may be difficult or impossible to drain safely, as attempts could pose a significant risk of complications such as pneumothorax (124). Additionally, KLAs often present as large, multiloculated abscesses with hypermucoviscous consistency that poses challenges for effective drainage using standard needle aspiration techniques (125, 126). Incomplete drainage can lead to persistent infections, an increased likelihood of complications such as the formation of new abscesses, bacteremia, metastatic infection, or even death (125).

**Fig 1:**
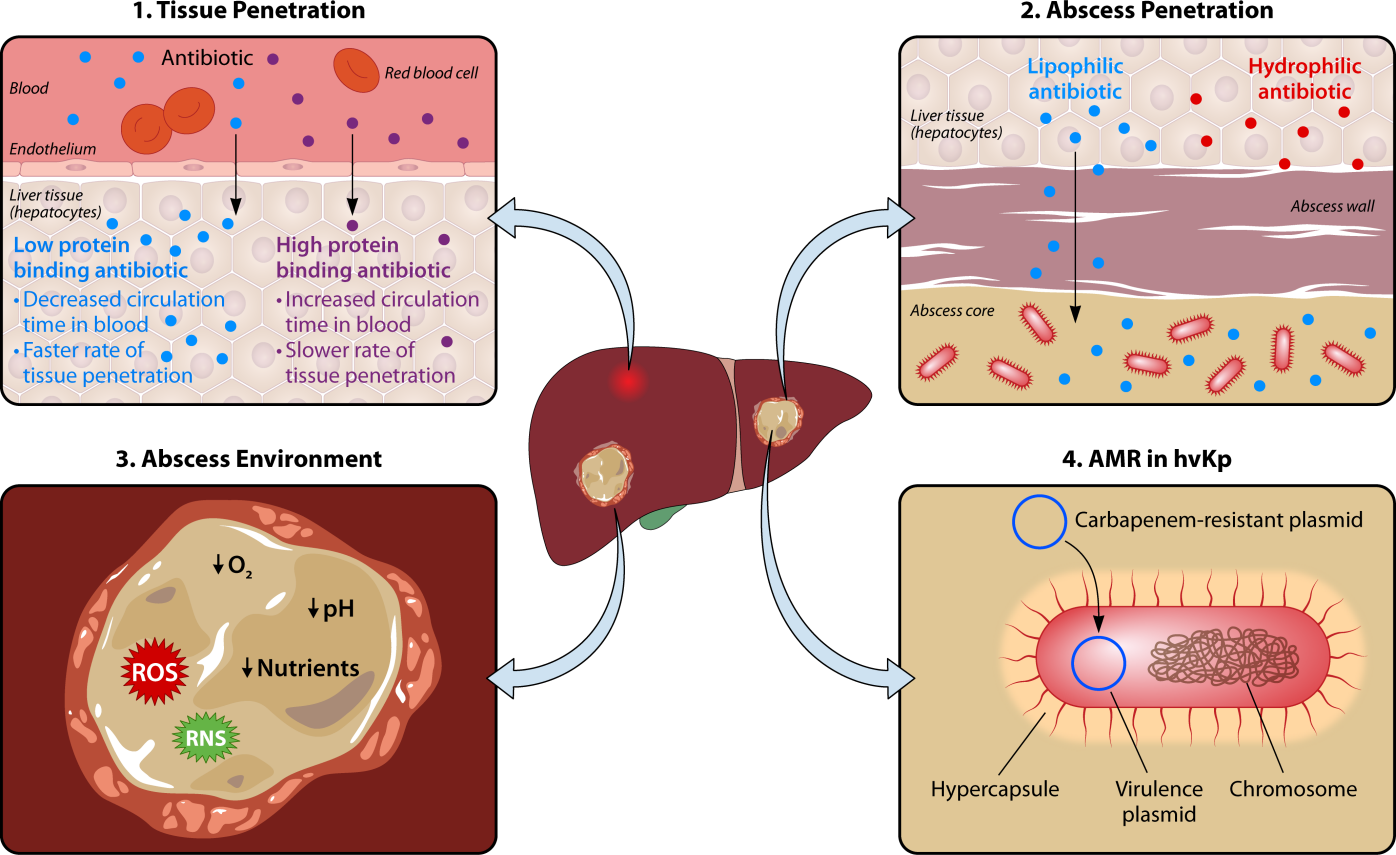
Barriers to effective antibiotic treatment of liver abscesses caused by hypervirulent *K. pneumoniae (hvKp)*. 1. Limited tissue penetration: For the treatment of KLAs, parenteral antibiotics are typically prescribed for rapid delivery to the bloodstream. However, there is limited blood supply to the abscesses, and so the antibiotic must travel from the bloodstream through the liver tissue and into the abscess. While antibiotics are in the bloodstream, they bind to plasma proteins. Different classes of antibiotic bind plasma protein to varying degrees, and only the unbound fractions can effectively penetrate tissue. Highly protein-bound antibiotics (such as third generation cephalosporins) penetrate tissues at a much slower rate than antibiotics with lower protein binding (such as fluoroquinolones and some carbapenems). 2. Abscess penetration: Abscess maturity and antibiotic lipophilicity determine the extent of antibiotic penetration through the abscess wall. Lipophilic (lipid soluble) antibiotics such as fluoroquinolones and macrolides are more effective at crossing the fibrous abscess wall than hydrophilic drugs like cephalosporins and aminoglycosides. 3. Abscess environment: Stresses encountered in the abscess environment, such as nutrient and oxygen deprivation, reactive oxygen or nitrogen species (ROS/RNS), and low pH are known to coerce bacteria into a low metabolic state, which increases tolerance to antibiotics. The extent to which these stresses contribute to treatment failure of KLAs has not been experimentally determined. 4. Antimicrobial resistance (AMR): The acquisition of genes or genetic mutations which confer resistance by hvKp reduces the repertoire of available antibiotics, limiting treatment options and increasing the risk of severe, recalcitrant infections.

Parenteral (non-oral) antibiotic therapy is generally preferred over oral administration to deliver the medication directly into the bloodstream. At present, no randomized clinical trials have specifically assessed the efficacy of various antibiotic regimens for treating KLAs. Consequently, the optimal antibiotic treatment for these infections remains a topic of ongoing debate (106, 108, 127). Historically, first-generation cephalosporins, with or without aminoglycoside therapy, were commonly selected due to their affordability, effectiveness, and the sensitivity of most hvKp strains that cause KLAs (106, 128). Cephalosporins combined with aminoglycosides offer synergistic bactericidal activity against certain Gram-negative infections; however, studies have not demonstrated improved clinical outcomes compared to cephalosporins monotherapy, for the treatment of KLAs (106, 108, 127). Additionally, the use of aminoglycosides carries a risk of nephrotoxicity, which may outweigh any theoretical benefit, particularly in critically ill patients. More recently, thirdgeneration cephalosporins such as ceftriaxone are used to treat KLAs due to their increased stability to β-lactamases and extended half-life for once daily dosing (51, 129). Additionally, ceftriaxone has a dual mechanism of elimination (through both the liver and kidney), which allows for treatment of patients with hepatic or renal dysfunction (129, 130). However, even when KLAs are treated with antibiotics deemed appropriate based on *in vitro* susceptibility testing, outcomes are inconsistent. In some cases, antibiotic therapy combined with drainage effectively resolved the KLA (112, 131, 132). In other cases, antibiotic therapy with drainage failed to resolve the abscess or fever, necessitating surgical removal of the infected liver tissue even in otherwise healthy individuals (125, 126, 133). The reasons for these contrasting outcomes are not well understood but could depend on a variety of factors including penetration of the drugs to the site of infection, antagonism of antibiotics by factors encountered at the infection site, or the metabolic state of the bacteria within the abscess (Fig. 1), described in more detail below.

### Tissue penetration

To effectively eliminate the bacterial infection, antibiotics must achieve a sufficiently high concentration and exhibit antimicrobial activity within the abscess (134). For an antibiotic to penetrate the abscess, it must first enter the bloodstream and cross the capillary endothelium, which separates the plasma from the surrounding tissue. The antibiotic then needs to diffuse through the interstitial fluid, pass through the abscess wall, and exert its antibacterial effect (135).

Capillary pores allow substances with a molecular weight of up to 1 kDa to pass through (135, 136). Antibiotics fall below this range, but plasma proteins like albumin, which bind to antibiotics, are too large to pass through these pores (135, 137). Albumin, synthesized primarily in the liver, is the most abundant protein in plasma, accounting for approximately 50%–60% of the total plasma protein content. Albumin-bound antibiotics have increased circulation time in the blood because the binding prevents their transport across capillary pores and into tissues. (Fig. 1) (135, 136). Fluoroquinolones (20%–40%) have relatively low protein binding compared to ceftriaxone which exhibits up to 95% protein binding (129, 138). Carbapenems display a broad range of protein binding, with ertapenem up to 94% protein-bound in contrast to meropenem which is only 2% protein-bound (139–141).

Critically ill patients, including those with KLAs, can experience hypoalbuminemia, a condition characterized by abnormally low levels of albumin in the blood (97, 142–144). Hypoalbuminemia can occur due to liver dysfunction or diabetes (145, 146). Reduced albumin levels in the plasma can increase the proportion of unbound antibiotics and, in theory, could enhance the tissue penetration of certain antibiotics. However, increased unbound antibiotic could also accelerate the elimination of the drug from the body, potentially diminishing their effectiveness. Several studies have suggested that antibiotic dosing, particularly for β-lactam antibiotics such as cephalosporins and carbapenems, may be suboptimal in critically ill patients due to hypoalbuminemia, leading to treatment failure or delayed therapeutic response (147–149). Modulating antibiotic dosing based on the unbound antibiotic fraction in patients with varying degrees of hypoalbuminemia may offer valuable insights and help optimize treatment, potentially improving outcomes in patients with KLAs.

### Abscess penetration

Although there have been limited studies on drug penetration into KLA abscesses, *in vivo* studies with bacterial abscesses at various anatomical locations have shown that antibiotic penetration into an encapsulated purulent abscess is restricted and largely influenced by the antibiotic class and the level of abscess maturation (135, 150). The ability of a drug to penetrate the abscess wall depends on the lipophilicity (lipid solubility) of the antibiotic (135, 150) (Fig. 1). Fluoroquinolones and macrolides have high lipophilicity and readily cross membranes, including abscess walls, more effectively than the hydrophilic β-lactams or aminoglycosides (135, 137, 138, 151). Human and animal pharmacokinetic data indicate that fluoroquinolones, effectively penetrate abscesses (135, 150). Interestingly, a recent study reported patients suffering with KLAs had shorter hospital stays and required less intravenous antibiotic therapy when treated with fluoroquinolones compared to those treated with β-lactams (152). The reasons for this are not known but could be at least partially attributed to the hydrophilic nature of β-lactams, which could limit their accumulation inside abscesses compared to lipophilic fluoroquinolones. While fluoroquinolones could be an effective treatment option for KLA infections, their effectiveness is increasingly compromised by rising rates of resistance, particularly in convergent strains (153, 154).

### Antibiotic activity within abscesses

Even if adequate antibiotic reaches the site of infection, the effectiveness of antibiotics in pus can be compromised by factors such as low pH and the metabolic state of the bacteria. Activity of aminoglycosides and certain fluoroquinolones diminishes in acidic environments, such as within liver abscesses, where the pH of the fluid typically ranges from 5.5 to 7.2 (135, 155, 156). Most conventional antibiotics are effective at killing actively growing bacteria as they typically target cellular processes such as cell wall synthesis or DNA replication (134, 157). For example, fluoroquinolones prevent the re-ligation step during DNA synthesis leading to double strand breaks and cell death in actively growing cells (158). Fluoroquinolones are, therefore, more effective against actively growing bacteria (158). β-lactam antibiotics (such as cephalosporins and carbapenems) target cell wall synthesis and, thus, are more effective against dividing cells that are actively synthesizing cell wall (159).

Antibiotic-tolerant cells are phenotypic variants in a population that are in a non-growing, metabolically quiescent state and can survive lethal doses of antibiotics without acquiring genetic resistance mechanisms (160, 161). Recent work has demonstrated that meropenem tolerance is widespread among clinical *K. pneumoniae* isolates (162). This tolerance is mediated by the formation of cell wall-deficient spheroplasts that survive prolonged β-lactam exposure and rapidly recover upon drug removal. Multiple contributing pathways, including cell envelope stress response systems (PhoPQ, Cpx, Rcs, and OmpR/EnvZ) that stabilize spheroplasts, while the lytic transglycosylase MltB counteracts the process (162). These findings reveal a mechanism of tolerance to carbapenems, an important class of antibiotics, and underscore the clinical relevance of non-resistant antibiotic tolerance in *K. pneumoniae*. Although the stresses that trigger antibiotic tolerance in *K. pneumoniae* during liver infection have not been explored, conditions which are known to induce antibiotic tolerance in other bacterial species (hypoxia [163, 164], nutrient limitation [165, 166], oxidative/nitrosative stress [167–170], acidic pH [135, 166]) are all detected during liver infection or within abscesses (135, 171–173). These stresses could similarly trigger antibiotic tolerance in KLAs (Fig. 1). Tolerant bacterial cells are not only difficult to eradicate but also serve as reservoirs for recurrent infections and for the development of resistance (174–177), underscoring the importance of understanding the precise nature of treatment failure against KLAs. Further research is required to understand the contribution of the antibiotic tolerance to treatment failure of KLAs.

## CONCLUSION

Underreporting of hvKp infections remains a significant concern, in part due to the absence of standardized, accessible diagnostic tools to differentiate hvKp from classical strains in Western healthcare settings. Currently, clinical management of *K. pneumoniae* bacteremia appropriately prioritizes identifying an anatomic source through imaging, such as CT or ultrasound, particularly in patients with persistent fever. Despite this, emerging evidence suggests that hvKp strains are disproportionately associated with deep-seated infections, such as liver abscesses, which may otherwise go unrecognized. While no inexpensive, rapid, and reliable test for hvKp is currently available, increased clinical awareness and targeted testing where feasible, especially in patients with no clear source of bacteremia, could facilitate earlier prognosis, help prevent the development of intractable liver abscesses, and ultimately improve patient outcomes.

Although still relatively rare, the emergence of convergent hvKp strains that combine hypervirulence with antibiotic resistance pose a serious public health threat, necessitating ongoing surveillance and rapid response strategies. Preventive efforts, including risk factor management, such as controlling diabetes and fatty liver disease, as well as potential decolonization strategies, may help reduce the incidence of KLA.

Despite major advances in the basic biology of hvKp pathogenesis, critical gaps remain in our understanding of hvKp abscess development, metastatic spread, and treatment failure. The complex interplay between bacterial virulence, host immune responses, and antibiotic penetration within abscesses is not yet fully understood, highlighting the need for continued research. Addressing these challenges will be key to improving both diagnostic capabilities and therapeutic approaches, ultimately enhancing patient outcomes.
